# Effects of neuromuscular electrical stimulation in patients with heart failure - review

**Published:** 2018

**Authors:** RL Ploesteanu, AC Nechita, D Turcu, BN Manolescu, SC Stamate, M Berteanu

**Affiliations:** * „Sf. Pantelimon” Clinical Emergency Hospital, Bucharest, Romania; ** “Carol Davila” University of Medicine and Pharmacy, Bucharest, Romania.; *** „Elias” Emergency Hospital, Bucharest, Romania; **** Faculty of Applied Chemistry and Materials Science, Bucharest, Romania University Politehnica of Bucharest, "C. Nenitescu” Organic Chemistry Department, Bucharest Romania

**Keywords:** neuromuscular electrical stimulation, NMES, heart failure, advanced heart failure, cardiac rehabilitation, oxidative stress

## Abstract

Research conducted in the last two decades suggests that neuromuscular electrical stimulation of the lower limb muscles (NMES) may be a „bridge” to conventional exercise or an alternative for patients with advanced chronic heart failure (CHF), non-compliant or non-responsive to physical training. Through stimulating the work of the skeletal muscles, NMES increases the functional capacity, muscle mass and endurance in patients with CHF. A beneficial effect of NMES on functional capacity, vascular endothelial function, quality of life and aerobic enzymes activity has been shown. A significant benefit of this novel therapy in heart failure is the fact that the procedure can be home-based, after prior guidance of the patient.

## Heart failure and oxidative stress

Heart failure (HF) is defined as a complex clinical syndrome that can result from any structural or functional cardiac disorder that impairs the ability of the ventricle to fill with or eject blood [**1**]. The major causes of HF are coronary artery disease, hypertension, cardiomyopathy, and valvular heart disease [**1**].

Following these pathologies, a process known as “cardiac remodeling” may occur. This pathophysiological remodeling of the heart involves changes in the structure and function of cardiac myocytes in the affected myocardium either directly or indirectly. These changes can lead to substantial alterations in the shape and volume of the heart and progressive ventricular dysfunction and clinically evident HF [**2**]. The mechanisms responsible for the development and progression of HF are the subject of rigorous investigation.

The substantial scientific research conducted on HF is due to its high mortality and prevalence, affecting approximately 1-2% of the adult population and rising to over 10% in patients aged over 70 years [**1**]. Over the past 20 years, significant development was noted in the treatment of HF by the introduction of multiple drugs (converting enzyme inhibitors, aldosterone receptor antagonists, beta blockers, angiotensin II receptor blocker neprilysin inhibitor) and cardiovascular procedures (cardiac resynchronization therapy). Nevertheless, despite the use of these modern methods of treatment, the latest European data show all-cause mortality at one year in hospitalized or outpatients with HF to be 17% and 7% and a readmission rate of 44% and 32%, higher in those with reduced left ventricle ejection fraction (LVEF) [**3**].

Given the poor prognosis of HF patients despite the use of multiple strategy-based treatments, scientists have been encouraged to search for new pathophysiological pathways that could be therapeutically targeted to the patient’s advantage including oxidative stress [**4**].

Oxidative stress represents an imbalance between the production of reactive oxygen species (ROS) and endogenous antioxidant defense mechanisms. These molecules are produced in the cells and play distinctive roles in both physiological signaling processes as well as pathological pathways when their production becomes impaired.

The cells constantly generate ROS, of which the principal molecule is the superoxide anion radical. Superoxide anions are synthesized as an unavoidable by-product of mitochondrial electron transport and enzymatically by xanthine oxidase. Other potential sources include prostanoid metabolism, catecholamine autoxidation, NAD (P) H oxidase activity, and the NO synthases [**5**].

Superoxide anions undergo spontaneous electron exchange reactions that induce the formation of hydrogen peroxide, hydroxyl radicals, and other redox-active derivatives.

The biological activity of ROS is opposed by a group of endogenous defense molecules called antioxidants. The most studied antioxidants are superoxide dismutase (SOD), glutathione peroxidase (GPX) and catalase. Important antioxidant nutrients are vitamins E, vitamin C and beta carotene.

Among the factors that can increase the production of ROS, we mention aging, muscle injury and inflammatory disease processes, including hyperthyroid myopathy, sepsis and HF.

Over the past several decades, oxidative stress has been involved in experimental and clinical studies involving HF patients. Markers of oxidative stress are elevated in CHF patients and have been associated with myocardial dysfunction, overall severity and poor prognosis of HF [**6**-**8**]. There are multiple proposed mechanisms through which oxidative stress might impair cardiac function and the first is by damaging cellular proteins and membranes, thus inducing cellular dysfunction or death through apoptosis and necrosis.

The superoxide anion is a potent inactivator of the signaling molecule NO that is involved in vascular endothelial dysfunction, with consequent loss of other physiological effects of NO [**9**]. Because ROS also modulate the activity of various intracellular signaling pathways and molecules, they can induce specific changes in proteins involved in myocardial excitation-contraction coupling [**4**]. ROS are involved in fibroblast proliferation and collagen synthesis, and also in matrix metalloproteinases (MMP) activation and increased expression [**10**]. Moreover, ROS activate a broad variety of hypertrophy signaling kinases and transcription factors and mediate apoptosis.

The importance of oxidative stress is increasing due to the involvement in the pathophysiological mechanism of cardiac remodeling responsible for the development and progression of HF [**11**].

The results of the study conducted by Kameda et al. showed that that myocardial oxidative stress is an important regulator of MMP activity and that this contributes to ventricular remodeling and left ventricular dilatation and dysfunction in patients with ischemic heart disease [**12**].

Kingery et al. concluded in a recent study that leukocyte iNOS is required for local and systemic inflammatory activation and cardiac remodeling in ischemic HF and that activated macrophages in HF patients may directly induce myocyte contractile dysfunction and oxidant stress [**13**].

Randomized clinical trials have been discouraging until now despite substantial pre-clinical data showing positive effects of reduced ROS activity by using antioxidant therapy in cardiovascular diseases. Vitamin E supplementation showed initial promise in observational studies but did not demonstrate any clinical benefits in the prevention or treatment of HF patients [**14**]. This can be explained, at least in part, by the difficulty of directly targeting ROS and thus minimizing the harmful effects on other physiological signaling pathways.

Recent research has shown that ROS signaling pathways are a complex system, and in many cases, essential for normal cardiovascular physiology, and that perturbation of these pathways resulting in dysregulated ROS is implicated in most cardiovascular diseases [**15**][**16**].

## Heart failure and cardiac rehabilitation

The main symptom of HF is the progressive decrease in functional capacity associated with dyspnea with prognostic implications independent of LVEF [**17**]. The pathophysiological process of HF will eventually lead to skeletal muscle weakness and atrophy, and when the symptoms will affect daily activities, to a sedentary lifestyle and social isolation with an impact on the prognosis of the patient. CHF-related skeletal muscle dysfunction is the result of an ongoing imbalance in the activation of anabolic and catabolic pathways [**18**] and has been shown to have significant prognostic importance [**19**].

Targeted patient management in HF consists of an integrated system which includes education programs, pharmacological, interventional therapy and cardiac rehabilitation programs. The European Society of Cardiology strongly recommends sustained and individualized physical activity in a standardized cardiac rehabilitation program [**1**]. Unfortunately, this recommendation is less enacted, recent studies showing that less than 20% of patients with HF participate in such a program [**20**].

In the meta-analysis carried out in 2004 [**21**], Piepoli et al. have shown in a total of 801 analyzed patients (395 received exercise training and 406 were controls) that exercise training leads to a reduction of 23% in the relative risk for death or hospitalization in HF patients. In the HF-ACTION large randomized control trial, after adjusting key prognostic factors, the investigators showed a reduction in cardiovascular mortality and hospitalization rate [**22**].

The physician coordinating the recovery must require active participation from the patient, be aware of the subject, explain the exercises and their effects, demonstrate exercises personally or by use of visual material, discuss with the patient, improve their mental status and continuously encourage the patient to use the examples of other patients who show improvements. The objectives of the cardiac rehabilitation program are to improve the clinical status by improving the symptoms which impeded exercise tolerance, to increase exercise capacity and psychological tonus and to prevent the aggravation of pathological heart condition.

The main component of cardiac rehabilitation in HF is physical exercise. Exercise therapy cannot be immediately used in all patients with severe HF symptoms, and so, in this situation, alternative therapies such as neuromuscular electrical stimulation (NMES), can probably show their highest benefits. Thus, while physical exercise will continue to serve as the major component of the rehabilitation programs, recent evidence demonstrating the potential benefits of adjunctive treatment options is collected.

## Effect of exercise training in heart failure patients on oxidative stress

Numerous studies, confirmed by meta-analyses, indicate that regular exercise training reduces cardiovascular mortality and events, coronary heart disease, heart failure and atherosclerosis [**23**-**29**].

Exercise and regular physical activity counteract the deleterious effects of aging, not only by combating sarcopenia, obesity and mitochondrial dysfunction, the major triggers of oxidative stress and inflammation in aging but also by exerting additional antioxidant and anti-inflammatory actions.

Cardiac rehabilitation through physical exercise is known to improve exercise tolerance, quality of life and decrease the rehospitalization rates of patients with HF [**30-32**].

It has also been proved that physical exercise has beneficial effects on endothelial dysfunction, neurohormonal activation, oxidative stress, inflammatory activation and depressive symptoms [**33**-**37**].

Anti-inflammatory effects have been reported in several interventional studies. Exercise therapy has been shown to reduce inflammatory markers, particularly CRP, TNF-*α*, interferon-gamma (INF-*γ*), monocyte chemoattractant protein-1 (MCP-1) interleukin-8 (IL-8), interleukin-18 (IL-18), sTNFR2, sTNF-R1, and soluble IL-6 receptor (sIL-6R), and to increase levels of anti-inflammatory factors such as IL-10, interleukin-12 (IL-12), interleukin-4 (IL- 4), and transforming growth factor beta 1 (TGF*β*1) [**34**, **38**-**40**].

It is worth highlighting that a minority of interventional and randomized controlled trials did not detect a significant effect of regular exercise on systemic inflammatory biomarkers in adults [**41**][**42**] or in aged adults [**43**]. Also, in the HF population, the same discrepancies remain, as some randomized trials did not show a significant decrease in inflammatory biomarkers in patients who received optimal medical therapy [**22**][**44**].

A meta-analysis found only five randomized controlled trials that examined the effects of regular aerobic exercise (of at least 4-week duration) in adults and concluded that aerobic exercise did not reduce C-reactive protein (CRP) levels [**45**]. The differences can be attributed to the smaller sample size used in the examined clinical trials.

The effect of resistance training on inflammation was the topic of research in several studies and the reported results were mostly negative [**46**-**48**]. However, Brooks et al. [**49**] reported that 16 weeks of resistance training reduced CRP and increased adiponectin levels in older diabetic patients.

Regular exercise improves age-associated oxidative stress in the heart [**50**], liver [**51**], plasma [**52**], arteries [**53**], and skeletal muscles [**54**][**55**]. In elderly people, regular exercise reduced serum/plasma levels of myeloperoxidase, a marker of inflammation and oxidative stress [**56**], and thiobarbituric-reactive acid substances, a marker of lipid peroxidation [**57**].

Visibly, the effects of exercise training on inflammation markers depend on the type (aerobic/resistance), intensity (mild/moderate/intense/exhaustive), frequency (sessions per day/week/month) of exercise, the subject’s main characteristics (age, sex and health or social condition) and therapy.

## Neuromuscular electrical stimulation and heart failure

NMES applied to leg muscles offers an alternative training mode and represents an attractive option for CHF patients who are unable, non-adherent or unwilling to exercise.

NMES consists of repeated, rhythmic stimulation of skeletal muscles in a static state, using skin electrodes positioned on the thighs and calf muscles, at an intensity that will lead to visible muscle contractions. The stimulator delivers a biphasic current of low frequency (10–25Hz), with gradually increasing stimulation amplitude of 40–80 mA maximized to the pain threshold of the subject.

NMES has been consistently shown to elicit positive effects on functional capacity and skeletal muscle adaptations in patients with HF and unable to participate in traditional aerobic and/or resistance training programs at an appropriate stimulus [**58**-**60**]. Prior reviews suggested that NMES produces similar improvement in a 6-min walk distance (6MWD) test, a simple test used to detect functional capacity, when compared to conventional aerobic exercise training used by cycle ergometer. Also, previous studies have shown that resistance training improved the distance in a 6-min walk test in patients with HF [**61**][**62**].

Peak oxygen uptake (peak VO_2_) is regarded as the gold-standard measurement of functional capacity. Reduced peak VO_2_, below 12 mL/kg/min, is associated with poor outcome independent of other risk factors in patients with HF [**17**]. Several studies have shown higher improvements in peak VO2 following NMES in HF patients with low functional capacity compared to those with average exercise capacity [**20**]. The benefits of NMES therapy in HF patients also occurred in an older age group (age 75 ± 4 years) [**63**].

Original investigations assessing the effects of chronic NMES programs in HF population are summarized in **Table 1**. In **Table 2**, the training protocol and major findings are reported.

**Table 1:** Summary of studies assessing NMES in patients with CHF.

Study, year	Protocol	Age (years)	Male (%)	NYHA class	LVEF (%)
**Maillefert, 1998 [**64**]** Randomized study	N=14	56.4±9.1	93	II, III, IV	22.3±8.8
**Vaquero, 1998 [**65**]** Randomized study	NMES (n=7) Control (n=7)	57±7	79	After orthotopic cardiac transplant	
**Harris, 2003 [**66**]** Randomized study	NMES (n=22) Cycle (n=24)	63±10 62±11	77 88	II, III	28.3±6.3 32.0±9.3
**Nuhr, 2004 [**67**]** Randomized study	NMES (n=15) Control (n=16)	53±7 53±13	93 82	II, III, IV	22±3 21±7
**Eicher, 2004 [**68**]** Randomized study	NMES (n=12) Cycle (n=12)	54±9	79	II, III	N/A N/A
**Deley, 2005 [**69**]** Randomized study	NMES (n=12) Cycle (n=12)	56±8 57±6	75 92	II, III	28.2±9.2 26.3±9.5
**Karavidas, 2006 [**70**]** Randomized study	NMES (n=16) Control (n=8)	57.4±15.3 63.8±8.1	87.5 87.5	II, III	27.5±6.5 27.2±4.5
**LeMaitre, 2006 [**71**]** Randomized study	NMES (n= 17) Cycle (n=19) healthy age-matched control (n=20)	63.9±2.6 60.7±2.6 61.4±4.7	71 79 55	II, III	28.7±1.7 32.4±2.7 N/A
**Deley, 2008 [**72**]** Randomized study	NMES (n=22) Cycle (n=22)	55±10 56±7	73 86	II, III, IV	23.7±7.4 23.2±10.6
**Karavidas, 2008 [**73**]** Randomized study	NMES (n=20) Control (n=10)	62±12 64±8	80 80	II, III	28±7 27±5
**Banerjee, 2009 [**74**]** Crossover study	NMES (n=10)	66	90	II, III	34
**Deftereos, 2010 [**75**]** Crossover study	NMES Cycle Total 31	60.7±2.1	77	II, III	30±3 29±1
**Araujo, 2012 [**76**]** Randomized strudy	NMES +conventional rehabilitation therapy (n=10) Control only conventional rehabilitation therapy (n=10)	52.2±9 42.5±14.3	60 60	II, III	37.6±6.9 38.3±8.8
**Dobsak, 2012 [**77**]** Randomized study	NMES (n=31) ET (n=30)	58.7±2.2 59.4±1.2	91 85	II, III	29.2±3.1 32.5±2.3
**Karavidas, 2013 [**78**]** Randomized study	NMES (n=15) Placebo (n=15)	69.4±8.6 68.5±7.9	67 67	II, III	63.6±7.6 62.6±4.5
**Labrunee, 2013 [**79**]** Crossover study	NMES (n=11) TENS (n=11)	54.4±3.8 62.7±3.6	64 64	III III	30±3.6 24±2.8
**Parissis, 2014 [**63**]** Randomized study	NMES (n=15) Placebo (n=15)	75.2±3.7 75.2±3.3	73 60	II, III	27.3±3.2 28±2.5
**Soska,** 2014 **[**80**]** Randomized study	NMES (n=23) NMES +AT (n=22) AT (n=26)	57.3±1.6 60.8±2.5 63.5±1.4	65 77 92	II, III	30.1±1.3 32.8±1.9 34.9±1.4
**Palau, 2016 [**81**]** Randomized study	NMES (n=15) NMES +IMT (n=15) IMT (n=15) Control (n=15)	68	70	II, III, IV	67
**Kadoglou, 2017 [**82**]** Randomized study	NMES (n=60) Placebo (n=60)	72±7 70±11	55 58.3	II, III	27.7±4.5 28.9±4.7
**Forestieri, 2017 [**83**]** Randomized study	NMES (n=24) Usual care group (n=25)	52.6±14.7 51.5±11	79 88	advanced HF stage D	27±4 25±3
**Iliou, 2017 [**84**]** Randomized study	ET+NMES group (n=50) ET (n=41)	57.6+9.8 59.2+7.2	76 78	II, III	31.9+4.4 30.4+6.7

IMT - inspiratory muscle training, TENS - transcutaneous electrical nerve stimulation, AT - aerobic training, ET - exercise training

**Table 2:** Training protocols and major findings.

Study	Training protocol	Major findings
**Maillefert, 1998**	Bilateral quadriceps and calf muscles 10 Hz, biphasic; Pulse duration: 200 μs On/off time: 20/20 s Intensity: Maximum tolerated by patient 60 min/d, 5 d/wk., 5 wk.	No adverse events reported Significant increase: Peak VO_2_ (13.9%), 6MWTD (9.5%), Gastrocnemius muscle volume (5.4%), Soleus muscle volume (6.0%), Cardiac output did not vary during NMES or improve significantly following the intervention
**Vaquero, 1998**	NMES: Bilateral quadriceps, 30–50 Hz, biphasic On/off time: 6–10/30–50 s Intensity: Maximum tolerated by patient 30 min/d, 3 d/wk., 8 wk. Control: No electrical stimulation	No adverse events reported Significant increase in the NMES group: Peak VO_2_ (9.1%) No change in the control group
**Harris, 2003**	NMES: Bilateral quadriceps, calf muscles, 25 Hz, biphasic On/off time: 5/5 s Intensity set by patient to achieve muscle contraction without joint movement or discomfort 30 min/d, 5 d/wk., 6 wk. Bicycle: 30 min/d, 5 d/wk., 6 wk., 70% of maximum HR	No adverse events reported Significant increase in the NMES group: Exercise time (13.4%), 6MWTD (8.1%), Quadriceps strength (12.5%), Quadriceps fatigue (14.3%); Significant increase in the bicycle group: Exercise time (20.2%), 6MWTD (9.0%), Quadriceps strength (10.9%), Quadriceps fatigue (10.5%), Peak VO_2_ did not improve in either group, Quality-of-life score improved for the entire study group (NMES and bicycle) but did not improve for each group independently Aforementioned improvements were not statistically significant between groups
**Nuhr, 2004**	NMES: Bilateral hamstrings and quadriceps, 15 Hz, biphasic On/off time: 2/4 s Pulse width: 0.5 ms Intensity: 25%–30% maximal voluntary contraction 4 h/d (2 AM; 2 PM), 7 d/wk., 10 wk. Control: Sensory electrical stimulation only	No adverse events reported Significant increase in the NMES group: Peak VO_2_ (20.8%), 6MWTD (31.7%), Myosin heavy chain isoforms shifted significantly toward the more oxidative type (19.4% increase) and away from more glycolytic, faster types (19.6% decrease), Citrate synthase activity (30.3%) No change or significant decrease in the control group in aforementioned variables
**Eicher, 2004**	NMES: Bilateral calf and quadriceps, 10 Hz, On/off time: 20/20 s 1 h/d, 7 d/wk., 25 d Bicycle: 20min/d, 7 d/wk., 25 d, 60-80% of maximum HR	No adverse events reported Significant increase in the NMES group: 6MWTD (18%), Peak VO_2_ (6%), blood flow velocity (42%) Significant increase in the bicycle group: 6MWTD (6%), Peak VO_2_ (9%), exercise duration (15%)
**Deley, 2005**	NMES: Bilateral quadriceps and calf muscles, 10 Hz, biphasic On/off time: 12/8 s Pulse duration: 200 μs Amplitude set to highest tolerable for the patient 60 min/d, 5 d/wk., 5 wk. Conventional exercise: Aerobic exercise (treadmill, bicycle and arm cycling) at 60–70% peak HR; target exertion by Borg scale 13–15, 60 min/d, 5 d/wk., 5 wk.	No adverse events reported Significant increase in the NMES group: Peak VO_2_ (8.2%), 6MWTD (11.9%), Maximal knee extensor isometric contraction at 90° (9.7%) Significant increase in the bicycle group: Peak VO_2_ (21.8%), 6MWTD (15.3%), Maximal knee extensor isometric contraction at 90° (11.3%) Aforementioned improvements were not statistically significant between groups
**Karavidas, 2006**	NMES: Bilateral quadriceps, calf muscles, 25 Hz, biphasic On/off time: 5/5 s Intensity: visible muscle contraction not strong enough to elicit discomfort or joint movement 30 min/d, 5 d/wk., 6 wk. Control: Sensory electrical stimulation only 30 min/d, 5 d/wk., 6 wk.	No adverse events reported Significant increase in the NMES group: 6MWTD (11.9%), Quality-of-life score (18.4%), TNFα (17.5%), sICAM-1 (15.6%), sVCAM-1 (13.1%), Baseline brachial artery diameter (2.0%), Hyperemic brachial artery diameter (3.5%), Flow mediated dilatation (29.6%) No change in the aforementioned variables in the control group Peak VO_2_ and LVEF did not significantly improve in either group
**LeMaitre, 2006**	NMES: Bilateral quadriceps, calf muscles, 25 Hz, biphasic On/off: 5/5 s 30 min/d, 5d/wk., 6 wk. Bicycle: 30 min/d, 5 d/wk., 6 wk., 70% of maximum HR	No adverse events reported Significant increase in the NMES group: Treadmill exercise time (s) (12%), 6MWTD (12%), Max quad strength (kg) (13%), Quadriceps fatigue index (17%); Significant increase in the bicycle group: Peak VO_2_ (16%), Treadmill exercise time (s) (27%), 6MWTD (13%), Max quad strength (kg) (13%), Quadriceps fatigue index (9%). Aforementioned improvements were not statistically significant between groups
**Deley, 2008**	NMES: Bilateral quadriceps and calf muscles, 10 Hz, biphasic On/off time: 12/8 s Pulse duration: 200 μs Amplitude set to highest tolerable to patient 60 min/d, 5 d/wk., 5 wk. Treadmill exercise: Heart rate corresponding to ventilatory threshold on baseline exercise test 60 min/d, 5 d/wk., 5 wk.	No adverse events reported Significant increase in the NMES group: Peak VO_2_ (12.2%), 6MWTD (13.8%) Significant increase in the bicycle group: Peak VO_2_ (16.7%), 6MWTD (16.5%) Aforementioned improvements were not statistically significant between groups The greatest improvements were realized by those with the lowest baseline exercise capacity in both groups
**Karavidas, 2008**	NMES: Bilateral quadriceps and calf muscles, 25 Hz, biphasic On/off time: 5/5 s Amplitude set to elicit a muscle contraction without discomfort or significant movement at knee or ankle joints 30 min/d, 5 d/wk., 6 wk. Control: Same NMES protocol but amplitude set to a level that did not elicit a muscle contraction	No adverse events reported Significant increase in the NMES group: 6MWTD (9.3%), Quality-of-life score (37.2%) No change in the aforementioned variables in the control group Nonsignificant trend toward a reduction in B-type natriuretic peptide only in the NMES group (6%, P =0.053)
**Banerjee, 2009**	NMES: Bilateral quadriceps, hamstrings, calf muscles, and gluteal muscles 4 Hz, rhythmic contraction Maximum current: 300 mA Intensity: 90% of heart rate reserve, determined individually 60 min/d, 5 d/wk., 8 wk. Washout phase: Return to habitual physical activity level	No adverse events reported but inability to tolerate NMES was the drop out cause for 2 patients Significant increase in the NMES group: Peak VO_2_ (10%), 6MWTD (9.6%), maximal knee extensor isometric contraction at 90° (7.1%) No significant difference in the aforementioned variables between baseline and washout. The greatest improvements were achieved by those with the lowest baseline exercise capacity and strength. No changes in LVEF and diastolic function.
**Deftereos, 2010**	NMES: Bilateral quadriceps and calf muscles, 25 Hz On/off time: 5/5 s 30 min/d, 5 d/wk., 6 wk. Bicycle: 30 min/d, 5 d/wk., 6 wk., 70 % of maximal HR	No adverse events reported Significant increase in the NMES group: 6MWTD (10%), Peak VO_2_ (6%), endothelial function FMD (38%), Endothelium-independent vasodilation (1.4%), Significant increase in the bicycle group: 6MWTD (13%), Peak VO_2_ (14%), endothelial function (48%), endothelium-independent vasodilation (2%). Significantly higher FMD value after bicycle training compared to NMES Significantly higher 6MWTD and Peak VO_2_ after bicycle training compared to NMES LVEF did not significantly improve in either group
**Araujo, 2012**	NMES: bilateral quadriceps 20 Hz, Pulse duration: 200 μs 60 min x 2/d, daily until hospital discharge Control: 60 min x 2/d, daily until hospital discharge but the electrostimulation device was turned off.	No adverse events reported Significant improvement in the NMES group: 6MWTD (127%), lactate decreased (33%) No change in the aforementioned variables in the control group
**Dobsak, 2012**	NMES: bilateral quadriceps and calf muscles, 10 Hz, biphasic On/off time:20/20 s Intensity: 60 mA 60 min x 2/d, 7d/wk., 12 wk. ET: 12 wk. total with bicycle: 40 min 2 wk., 20 min in the last 10 wk. and resistance training 20 min last 10 wk.	No adverse events reported Significant beneficial effects in the NMES group: Peak VO_2_ (9.8%), Big-endothelin pmol/L (-25%), CRP mg/L (-65.3%) Significant beneficial effects in the aerobic ET group: Peak VO_2_ (11.2%), Big-endothelin pmol/L (-8.2%), CRP mg/L (-60%) Aforementioned improvements were not statistically significant between groups No changes in LDL, HDL and glucose level Positive effect after 12 weeks of ET or NMES on arterial stiffness and autonomic balance in patients with moderate CHF
**Karavidas, 2013**	NMES: Bilateral quadriceps and calf muscles, 25 Hz On/off time: 5/5 s Intensity: visible muscular contraction 30 min/d, 5 d/ wk., 6 wk. Placebo: same regimen, 5 Hz, without visible muscular contractions	No adverse events reported Significant beneficial effects in the NMES group: 6MWTD (23.8%), FMD (73.6%), improvement in quality of life and depression assessed by KCCQ, MLHFQ scores, BDI questionnaires and Zung self-rated depression scores. Placebo group: no change in FMD, A tendency toward a lower mitral E/e’ wave ratio was observed in the NMES group Significant difference between groups: FMD, 6MWTD, quality of life and depression BNP nonsignificant change in plasma BNP levels was observed between both groups
**Labrunee, 2013**	NMES: left leg quadriceps and triceps surae muscle, 25 Hz Duration: 5 min On/off time: 3/3 s TENS: left leg quadriceps and triceps surae muscle Current non polarized, 80 Hz, Duration: 5 min On/off time: 3/3 s cross over, randomized and sham controlled	No adverse events reported Significant beneficial effects in the NMES group: reduce MSNA Significant beneficial effects in the NMES group: reduce MSNA No variation of blood pressure, heart rate or respiratory parameters was observed after stimulation
**Parissis, 2014**	NMES: Bilateral quadriceps and calf muscles, 25 Hz On/off time: 5/5 s Intensity: visible muscular contraction to pain threshold 30 min/ d, 5 d/ wk., 6 wk. Placebo: Bilateral quadriceps and calf muscles, 5 Hz (did not lead to palpable contractions 30 min/ d, 5 d/ wk., 6 wk.	No adverse events reported Significant beneficial effects in the NMES group: FMD (120%), KCCQ, MLHFQ scores, BDI questionnaires and Zung self-rated depression scores. Significant difference between groups: FMD, quality of life and depression
**Soska, 2014**	NMES: bilateral extensors muscle, 10 Hz, On/off time: 20/20 s 60 min x 2/ d, 7 d/wk., 12 wk. AT: Bicycle, 10 min+40 min +10 min, 3x/wk., to individual anaerobic threshold, first 2 wk. AT 20 min and resistance training 20 min for the following 10 wk. AT + NMES: identical AT + identical NMES, 12wk	No adverse events reported Significant beneficial effects in the NMES group: Peak VO_2_ 8.3%), Duration of exercise min (9.4%), quality of life MLHF score (-16.6%) Significant beneficial effects in the AT group: Peak VO_2_ (15.2%), Duration of exercise min (19.8%), quality of life MLHF score (-27.9%) Significant beneficial effects in the AT+NMES group: Peak VO_2_ (15.3%), Duration of exercise (min) (10.7%), quality of life MLHF score (-29.1%) The results of the three studied rehabilitation training protocols did not significantly differ statistically. It can be stated that aerobic ET combined with EMS adds no statistically significant benefit
**Palau, 2016**	NMES: bilateral quadriceps and gastrocnemius muscles, 10-50 Hz On/off time: 5/5 s Intensity: pain threshold 45 min/d, 2 d/wk., 12 wk. IMT: 20 min x 2/d, 7 d/ wk., 12 wk. IMT + NMES: identical IMT + Identical NMES Standard treatment: no IMT, NMES	Ongoing
**Kadoglou, 2017**	NMES: Bilateral quadriceps and gastrocnemius muscles, 25 Hz On/off: 5/5 s Intensity: visible muscular contraction 30 min/d, 5 d/ wk., 6 wk. Placebo: 5 Hz, not leading to a visible or palpable contraction	No adverse events reported Significant beneficial effects in the NMES group: 6MWT, hospitalization rate. Patients after NMES had no difference compared to non-NMES patients in terms of survival The hospitalization rate was significantly lower in the NMES group before and after adjustment for major prognostic factors
**Forestieri, 2017**	NMES: bilateral quadriceps and calf muscles, 40 Hz, On/off: 10/20 s Intensity: visible muscular contraction 60 min x2/day, daily, 2 wk. Control: breathing exercises and global active exercises of the upper and lower limbs in bed	No adverse events reported Stimulation group exhibited a significantly higher increase compared to the control group in terms of 6MWT. NMES group: significantly higher dose reduction of dobutamine compared to the control group
**Iliou, 2017**	NMES+ET: 20±5 low frequency NMES for quadriceps muscles after aerobic training and/or additional physical activities, 10Hz biphasic current, Pulse duration 200μs, On/off: 20/40 s, ET: 20±5 physical training sessions, 4–8 weeks Session: 30–60-minute period of aerobic exercise training on a bicycle or treadmill	NMES on top of ET does not demonstrate any significant additional improvement in exercise capacity in moderately severe and stable CHF patients.

HR- heart rate, FMD - flow mediated dilatation, MSNA - Muscle Sympathetic Nerve Activity, ET – exercise therapy

## Neuromuscular electrical stimulation and oxidative stress in HF patients

From the previous studies presented in this review only the following presented data regarding the effects of NMES on inflammation and oxidative stress.

Dobcek et al. [**77**] concluded that exercise training or NMES significantly decreases the plasmatic level of big-endothelin (-18% in average) and CRP (-62% in average), showing anti-inflammatory effects.

Karavidas et al. [**70**] showed that circulating cytokine TNF-α and the IL-10/TNF-a ratio were significantly modified after the NMES program in favor of the anti-inflammatory action, whereas their control group remained unaffected. In this study, there was a significant reduction in the circulating cellular adhesion molecules (soluble ICAM-1 and soluble VCAM-1) in the NMES group indicating an NMES-induced beneficial modulation of endothelial–monocyte cellular interactions in chronic heart failure.

Nuhr et al. [**67**] found that the levels of citrate synthase (CS) and glyceraldehydes phosphate dehydrogenase (GAPDH), enzymes that indicate the citric acid cycle and glycolysis activity, displayed significant changes through the NMES program. CS activity increased whereas GAPDH activity decreased. In the control groups, neither CS nor GAPDH were significantly changed using sham stimulation. During follow-up, the groups differed significantly in terms of CS and GAPDH.

The findings in the study of Vaquero et al. [**65**] are in agreement with previous research which has shown that NMES combined with muscle training might induce an increase in the oxidative capacity of human muscle (in the activity of succinate dehydrogenase) even greater than that induced by muscle training alone.

It appears that, in fact, NMES has beneficial effects on inflammation and oxidative stress, both of these mechanisms with implications in the pathophysiological mechanism of HF progression.

## Conclusion

To improve the poor prognosis of patients with HF we need to develop therapeutic strategies based on a novel understanding of the pathophysiology of HF. The NMES therapy showed a beneficial effect in a specific type of HF population with advanced disease, an area where the physician and the patient need the most help. The approach of regulating oxidative stress throughout NMES therapy may contribute to establishing effective treatment strategies for HF.

## Conflict of interest:

The authors declare that there is no conflict of interest.
